# Case Report: Interleukin-23 blockade achieves sustained remission in Niemann–Pick type C-associated Crohn’s disease refractory to anti-tumor necrosis factor therapy

**DOI:** 10.3389/fimmu.2026.1766960

**Published:** 2026-02-19

**Authors:** Toshihiko Kakiuchi, Satomi Shimada, Yumeng Zhang, Miri Nomura, Masato Yoshiura

**Affiliations:** Department of Pediatrics, Faculty of Medicine, Saga University, Saga, Japan

**Keywords:** biological products, Crohn disease, granuloma, interleukin-23, Niemann–Pick disease type C

## Abstract

**Background:**

Niemann–Pick type C (NPC) is a lysosomal lipid trafficking disorder that is associated with defective autophagy and impaired bacterial clearance, potentially predisposing patients to Crohn’s disease (CD)-like granulomatous enteritis. Tumor necrosis factor-alpha inhibitors are typically the first-line biologic treatment for CD. However, due to limited evidence-based data, the optimal treatment for NPC-associated CD remains unclear.

**Case presentation:**

A 20-year-old female patient with genetically confirmed NPC developed diarrhea and refractory perianal abscesses at the age of 17 years. Colonoscopy showed discontinuous longitudinal erosions, and histological examination revealed chronic inflammation with granulomas. Based on these finding, a diagnosis of CD complicating NPC was made. Adalimumab was not successful in maintaining remission despite corticosteroid therapy. Ustekinumab induced steroid-free remission. Nevertheless, its effect diminished before each dosing interval. Switching to risankizumab achieved stable remission, which was maintained for 3 years. During interleukin (IL)-23 blockade therapy, the patient developed an infection associated with an intrathecal baclofen pump.

**Conclusion:**

This case indicates that IL-12/23 and IL-23 inhibitors can be effective therapeutic options for NPC-associated CD. Furthermore, it supports the hypothesis that IL-23-driven inflammation plays a role in the development of this condition. These findings should be considered hypothesis-generating and validated in additional cases.

## 1 Introduction

Niemann–Pick type C disease (NPC) is an incurable cholesterol-storage disorder that is caused by inherited deficiencies of lysosomal proteins involved in intracellular lipid trafficking. Mutation in the *NPC1* or *NPC2* gene is an underlying cause (1). The condition manifests as progressive neurological impairment and leads to death at an early age (2). Crohn’s disease (CD) is a chronic inflammatory bowel disease (IBD) of unknown etiology. Granulomas are nodular lesions composed primarily of macrophages that form due to a chronic immune response. In CD, solitary non-caseating granulomas form against the background of transmural inflammation, from the intestinal mucosa to the serosal membrane (3). The two conditions may be unrelated at first glance. However, NPC1 mutations cause impaired bacterial clearance via abnormalities in the macrophage/autophagy pathway, resulting in CD-like early-onset granulomatous enteritis (4).

Biological agents are used for the treatment of CD. Among them, tumor necrosis factor-alpha (TNF-α) inhibitors play a major role (5). Various biological agents are currently being developed for CD. However, the treatment guidelines for pediatric CD recommend TNF-α as the first-line biological agent (6). However, there is limited information about the treatment of NPC-associated CD, and there are still no clear recommendations. Williams et al. (7) have reported that anti-TNF-α therapy is safe and may be an effective therapeutic strategy for managing intestinal inflammation in in patients with NPC.

Herein, we present a 20-year-old female patient with NPC who was diagnosed with CD at the age of 17 years after experiencing diarrhea and refractory perianal abscesses. An interesting immunological point is that the introduction of adalimumab was not effective. However, treatment with ustekinumab (UST), a selective interleukin (IL)-12 and IL-23 p40 subunit inhibitor, and risankizumab (RZB), a selective IL-23 p19 subunit inhibitor, was successful.

## 2 Case description

This female patient was diagnosed with NPC at birth, and she continually underwent outpatient follow-up at our hospital’s pediatric department. She was born via emergency cesarean section at 34 weeks of gestation due to progressive cardiac enlargement and hepatosplenomegaly. After birth, she underwent bone marrow examination, which revealed foamy cells, and filipin staining showed the presence of fibroblasts. Based on these findings, a diagnosis of NPC was established. Subsequent genetic testing identified a compound heterozygosity for a known pathogenic variant. At approximately 3 years of age, rapid regression in psychomotor development occurred. Intravenous infusion of β-cyclodextrin was started at age 4 years, miglustat at age 5 years, and intravenous administration of 2-hydroxypropyl-β-cyclodextrin from Ommaya reservoir at age 6 years. At 8 years of age, an ITB pump was implanted in the patient’s abdomen for intrathecal baclofen therapy for spasticity. By the age of 10 years, she required laryngotracheostomy and gastrostomy, and both procedures were then performed.

**Figure 1:**
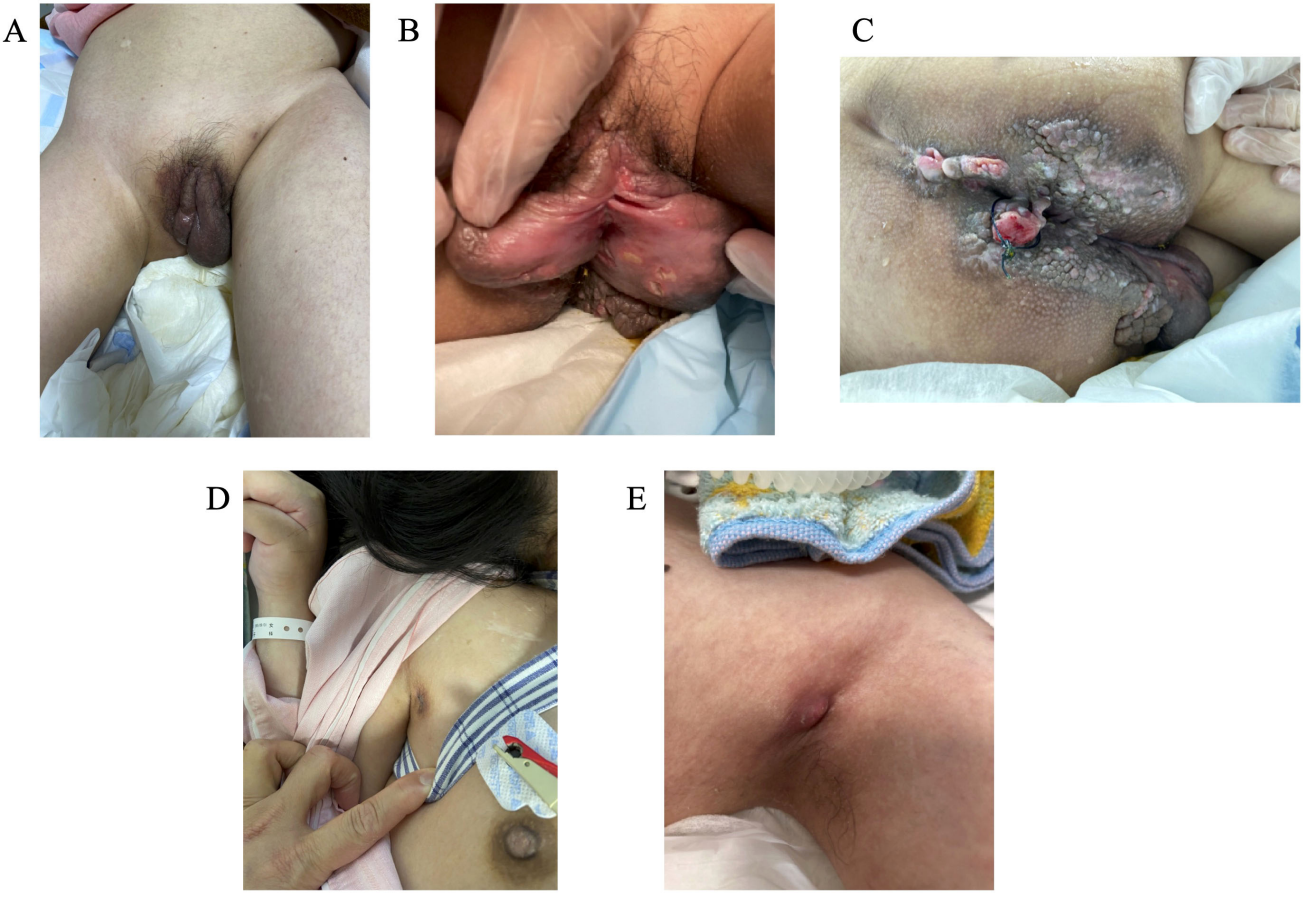
The patient had swelling and erosions in the genital area **(A, B)**, mainly in the labia majora, perianal diseased granulation tissues and abscesses **(C)**, and an abscess in both axilla **(D, E)**.

**Figure 2:**
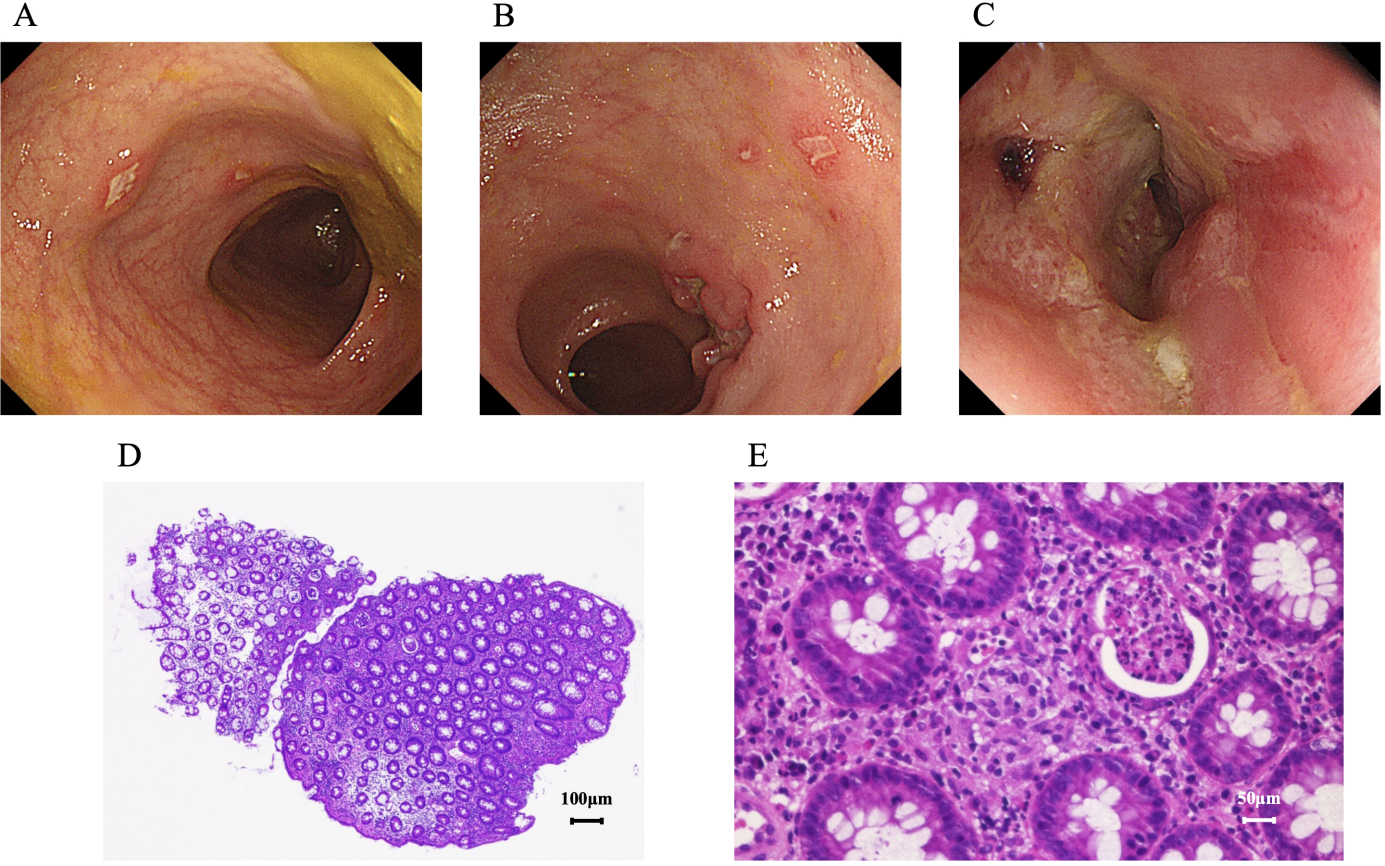
Her second total colonoscopy revealed erosions in the transverse colon **(A)**, ulcers and longitudinal erosions in the descending colon **(B)**, and circumferential mucosal redness and erosions in the rectum **(C)**. Pathological examination of the mucosa taken from the ulcer site revealed inflammatory cell infiltration (D, hematoxylin and eosin staining, scale bar = 100 μm) and the formation of non-caseating granulomas (E, hematoxylin and eosin staining, scale bar = 50 μm).

Swelling and erosions of the genital area, mainly the labia majora, and perianal diseased granulation tissues and abscesses were noted at approximately age 14 years (**Figures 1A–C**). Simultaneously, a small area of skin swelling was observed in both axilla, and there was pus discharge from that area (**Figures 1D, E**). The Seton procedure was carried out. However, there was no improvement in the patient’s condition. Initial esophagogastroduodenoscopy and total colonoscopy (TCS) at age 14 years revealed no abnormalities, and symptomatic treatment was continued. A second TCS at the age of 17 years revealed discontinuous ulcers and erosions. The erosions showed a longitudinal tendency (**Figures 2A–C**). Pathological examination of the gastrointestinal mucosa revealed inflammatory cell infiltration (plasma cells and lymphocytes), fibrosis, cryptitis, and the formation of pseudopapillary granulomas (**Figures 2D, E**). Her leucine-rich alpha 2 glycoprotein (LRG, normal range <16.0 µg/mL) level had risen to 48 µg/mL. Based on these results, a diagnosis of CD complicating NPC was made.

**Figure 3:**
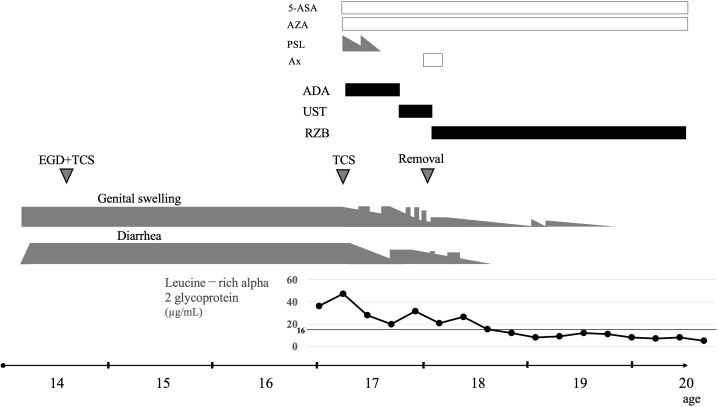
Clinical course of Niemann–Pick type C disease-associated Crohn’s disease. 5-ASA, 5-aminosalicylic acid; AZA, azathioprine; PSL, prednisolone; Ax, antibiotics; ADA, adalimumab; UST, ustekinumab; RZB, risankizumab; EGD, esophagogastroduodenoscopy; TCS, total colonoscopy.

Her clinical course is shown in **Figure 3**. Treatment with 5-aminosalicylic acid and azathioprine was initiated. However, considering the severity of the patient’s diarrhea, vulvar swelling (particularly in the labia majora), and perianal disease, advanced therapy was required. According to the current treatment guidelines for CD (6), anti-TNF-α agents were considered. Although infliximab was the first-line therapeutic option, it became evident that securing peripheral venous access would be challenging in her case. Therefore, adalimumab, which can be administered subcutaneously, was selected. Prednisolone (PSL) was added during the induction phase with adalimumab, leading to an initial clinical response. Her LRG levels normalized in proportion to the improvement in her clinical symptoms. Nevertheless, her condition clearly worsened as the PSL dose was tapered, and remission could not be maintained with adalimumab. The treatment was subsequently switched to UST. Because UST is initially administered intravenously and subsequently given subcutaneously every 8 weeks, it was considered feasible in this case.

After switching to UST, the patient achieved and maintained remission without PSL. However, after approximately 6 months, although diarrhea and vulvar swelling (particularly in the labia majora) improved immediately after each UST injection, her symptoms began to recur at approximately week 6 of the dosing interval. As the therapeutic effect of UST could no longer be sustained for the full 8 weeks, the regimen was changed to RZB. The first 3 doses of RZB were administered intravenously every 4 weeks, followed by subcutaneous injections every 8 weeks thereafter, making it a feasible option. After switching to RZB, the patient maintained remission with the 8-week dosing schedule, which was in contrast to her experience with UST. Her LRG levels rose temporarily after UST but normalized relatively quickly with the introduction of RZB. She has now remained in stable remission without diarrhea or vulvar swelling for 3 years since initiating RZB therapy.

When a change in treatment for CD from UST to RZB was being considered, the patient presented with temperature fluctuations and tachycardia. Her cerebrospinal fluid sample collected from the Ommaya reservoir showed a mild increase in white blood cell count, and *Roseomonas mucosa* was identified on culture. Although UST was used, the severity of NPC symptoms worsened, thereby reducing the need for the ITB pump and intraventricular cyclodextrin administration. Pump removal and antibiotic treatment (switching the initial cefepime to minomycin) rapidly resolved the symptoms, leading to a complete recovery from ventricular meningitis.

## 3 Discussion

Previous reports describing the therapeutic strategies for CD in patients with NPC are extremely limited, and TNF-α therapy has been considered appropriate based on previous case reports (7, 8). Williams et al. have reported that anti-TNF-α agents are associated with favorable outcomes in NPC-associated CD-like disease (7). However, the findings of our case are in contrast to these earlier findings: adalimumab was ineffective, and UST, an IL-12/23 p40 inhibitor, and RZB, an IL-23 p19 inhibitor, induced and sustained remission. To the best of our knowledge, this is the first case report showing the superiority of IL-12/23 or IL-23 inhibitors over TNF-α inhibitors in NPC-associated CD.

Classical CD is challenging to distinguish from NPC-associated enteropathy. In this patient, discontinuous longitudinal erosions, transmural inflammation, and non-caseating granulomas supported a diagnosis of CD rather than of nonspecific NPC-related enteropathy. A comprehensive monogenic IBD panel testing was not performed. However, the patient had genetically confirmed NPC, which itself represents a monogenic disorder that predisposes to CD-like granulomatous enteritis. No additional extraintestinal manifestations indicative of alternative monogenic IBD were observed.

The unique immunological abnormalities arising from NPC can be a possible explanation for this distinctive treatment response. NPC1 deficiency disrupts lysosomal lipid trafficking and autophagic flux, leading to defective bacterial clearance and altered innate immune activation (9, 10). Autophagy dysfunction is recognized as a key pathogenic mechanism in the development and progression of CD (11). Genetic variants in autophagy-related genes, including *ATG16L1*, *IRGM*, and *NOD2*, impair intracellular bacterial handling and antigen processing in intestinal epithelial and Paneth cells, leading to dysregulated immune responses to the gut microbiota and persistent intestinal inflammation (12). In addition, defective autophagy in macrophages and dendritic cells contributes to abnormal cytokine production and disruption of intestinal immune homeostasis (13). In the present case, macrophage polarization (M1/M2) was not directly evaluated; therefore, definitive conclusions cannot be drawn. However, autophagy dysfunction has been reported to promote polarization toward pro-inflammatory M1 macrophages and to contribute to persistent inflammation (14). Given that NPC is characterized by intrinsic lysosomal and autophagy impairment, such mechanisms may have disrupted intestinal immune homeostasis and contributed to both the development and clinical course of CD in this patient. In NPC, macrophages exhibit enhanced inflammasome activity and increased production of pro-inflammatory cytokines, including IL-1β and IL-23 (9). The IL-23/Th17 axis is important for sustaining chronic granulomatous inflammation (15), particularly in very-early-onset and monogenic types of IBD (16). Hence, NPC-associated enteritis may be caused predominantly by IL-23-dependent mechanisms rather than TNF-α-mediated pathways.

The clinical course of our patient supports this hypothesis. UST improved symptoms. However, it did not maintain the full dosing interval effect. Meanwhile, RZB, which selectively blocks IL-23p19, provided stable remission. This finding is in accordance with accumulating evidence indicating superior and more durable disease control with IL-23-specific inhibition compared with IL-12/23-targeted therapy in specific CD phenotypes (17). The diminished durability of response to ustekinumab in this case should not be interpreted solely from a mechanistic perspective. Alternative explanations, such as suboptimal drug exposure, pharmacokinetic variability, dosing interval limitations, and immunogenicity, might also have contributed to the observed loss of response. Hudson et al. (18) reported a case of CD complicated by NPC in which infliximab (an anti–TNF-α agent) and UST were ineffective as monotherapies, whereas combination therapy resulted in clinical improvement. Similarly, in our case, neither ADA nor UST was effective when used alone; however, the possibility that their combination might have been effective cannot be excluded. Nevertheless, given the increased risk of complications and higher medical costs associated with dual biologic therapy in CD, our current treatment strategy prioritizes the use of a single biologic agent, particularly in light of the availability of selective interleukin-23 p19 subunit inhibitors.

Granuloma formation, a characteristic shared by NPC-associated intestinal inflammation and CD, further implicates IL-23 signaling. Dysregulated macrophage autophagy and defective bacterial handling in NPC likely promote granulomatous responses maintained by IL-23-driven Th17 activity (19, 20). Similar immunological patterns are observed in monogenic granulomatous diseases, such as chronic granulomatous disease and IL-10 receptor deficiency, where the IL-23/Th17 pathways play major pathogenic roles (21, 22).

Taken together, our findings indicate that NPC-associated CD may represent an immunologically distinct phenotype in which IL-23-dependent pathways are prominent. However, conclusions drawn from a single case should be interpreted with caution, and further studies with additional cases must be conducted before any general treatment recommendations can be made.

IL-12/23 inhibitors (e.g., UST) and IL-23-specific inhibitors (e.g., RZB) generally have favorable safety profiles. However, clinicians should remain aware of potential infectious complications. These agents regulate Th1–Th17–axis immunity, and they may reduce host defense capacity against opportunistic bacteria, mycobacteria, and fungal pathogens (17, 22). Large-scale clinical trials including adults and adolescents have reported low rates of major infections (23, 24). However, real-world experiences have shown that the risk increases if patients have structural vulnerabilities or indwelling medical devices.

In the current case, the patient developed an infection associated with an intrathecal baclofen pump and catheter during IL-23 inhibitor therapy. Causality could not be definitively established. However, IL-23 pathway inhibition may theoretically impair the inflammatory response essential for clearing biofilm-forming pathogens on implanted devices. Intrathecal pumps are susceptible to colonization by *Staphylococcus aureus* and other organisms, and immunomodulatory therapy may further compromise device-related immunity (25).When initiating treatment with IL-23 inhibitors in patients with implantable devices, heightened surveillance for early infectious signs and close multidisciplinary coordination are required. In the current case, the temporal association between IL-23 inhibition and device-related infection does not establish causality and should be interpreted as hypothesis-generating. Further studies should be carried out to validate whether IL-23 blockade influences the risk of infection in patients with implanted medical devices.

## Data Availability

The original contributions presented in the study are included in the article/supplementary material. Further inquiries can be directed to the corresponding author.
